# Lifestyle causal beliefs are associated with higher personal and perceived stigma regarding depressive disorders: results from a representative population survey

**DOI:** 10.1186/s12888-023-04907-5

**Published:** 2023-06-08

**Authors:** Katharina Scholze, Hanna Reich, Phyllis Passow, Christian Sander, Andreas Czaplicki, Ulrich Hegerl

**Affiliations:** 1 Depression Research Centre, German Depression Foundation, Leipzig, Germany; 2 Depression Research Centre of the German Depression Foundation, Department of Psychiatry, Psychosomatic Medicine and Psychotherapy, University Hospital, Goethe University, Frankfurt am Main, Germany; 3 Department of Psychiatry and Psychotherapy, University of Leipzig Medical Center, Leipzig, Germany; 4 Johann Christian Senckenberg Distinguished Professorship, Goethe-University, Frankfurt am Main, Germany

**Keywords:** Depression, Stigma, Aetiology, Personal contact with depression

## Abstract

**Background:**

Depression is a prevalent and severe disorder associated with considerable stigma. This stigma contributes to the suffering and impedes help seeking behaviour of those affected. Stigma can be influenced by causal beliefs about depression and personal contact with people suffering from depression. The aim of this study was to investigate (1) the associations between beliefs about the aetiology of depression and personal / perceived stigma, as well as (2) a possible moderating effect of personal contact with people with depression on these associations.

**Methods:**

Stigma, causal beliefs, and contact with depression were assessed in a representative online survey among German adults (N = 5,000). Multiple regression analyses were performed with contact levels (unaffected vs. personally affected (diagnosed) vs. personally affected (undiagnosed) vs. affected by relatives with depression vs. persons who treat depression) and causal beliefs (biogenetic vs. psychosocial vs. lifestyle) as predictor variables for personal and perceived stigma as dependent variables.

**Results:**

Higher personal stigma was associated with lifestyle causal beliefs (*p* < .001, *f²* = 0.07), lower personal stigma with biogenetic (*p* = .006, *f²* = 0.01) and psychosocial (*p <* .001, *f²* = 0.02) causal beliefs. A positive interaction between psychosocial beliefs and the contact group “relatives” (p = .039) further suggests that this contact group does not benefit so strongly from psychosocial causal beliefs regarding personal stigma. Higher perceived stigma was associated with psychosocial (*p* < .001, *f²* = 0.01) and lifestyle (*p* < .011, *f²* = 0.01) causal beliefs. Regarding contact levels, the “unaffected” had significantly higher personal stigma scores than each of the other contact groups (*p <* .001). The contact group “affected (diagnosed)” had significantly higher perceived stigma scores than “unaffected”.

**Conclusions:**

The available data show that anti-stigma campaigns should clearly communicate, that depression is not caused by an unfavorable lifestyle. In general, psychosocial or biological explanatory models should be explained. Especially for the target group “relatives of depressive patients”, who can be an important support for patients, education about biogenetic explanatory models should be provided. However, it is important to note that causal beliefs are only one of many factors that impact on stigma.

**Supplementary Information:**

The online version contains supplementary material available at 10.1186/s12888-023-04907-5.

## Background

Depression is a severe illness [1] with a high prevalence across countries [2, 3]. During the course of their depressive illness, many patients experience stigmatisation [4, 5].

Stigma is multidimensional and includes aspects such as attributing responsibility or blaming others or oneself for the illness, the impression that affected people could be dangerous and unpredictable [6] as well as the desire for social distance from affected individuals and discrimination [7]. Stigma is associated with lower help-seeking behaviour and lower use of mental health care services [8–11], which is one of the factors contributing to the large treatment gap in individuals with depressive disorders [12].

Depression stigma can be considered from three different perspectives:

1. *Personal depression stigma* - negative attitude of a person, affected or not affected, towards people suffering from depression [7].
2. *Perceived depression stigma* - a person’s assumption of how other people feel about people suffering from depression [7].
3. *Self-stigma* - occurs when affected people internalise the perceived stigma and apply it to themselves [13]. This can lead to lower levels of hope, empowerment, self-esteem, self-efficacy, quality of life, and social support [14].

Beliefs about the aetiology of depression can influence public views of depressed people [15, 16] as well as the way those affected view themselves [17], and thus stigma. One approach to reducing stigma is to educate people about the aetiological explanations of mental illness [18]. However, there is no consensus in the literature about how exactly beliefs about the aetiology of depression affect stigma.

According to the attribution theory, biogenetic causal beliefs should decrease the view that affected individuals are responsible for their condition and that they are to blame for the associated disease. Because attributing low responsibility to a stigmatising condition leads to less blame and more positive emotions (e.g., compassion, likeability and acceptance instead of anger) [19, 20]. Indeed, empirical findings suggest that biogenetic explanations are significant associated with higher social acceptance [21] and less personal stigma [22]. On the other hand, the diminished sense of control may enhance a different component of stigma, i.e. unpredictability and dangerousness [23]. Several studies suggest significant positive relations between biogenetic causal models and stigmatising attitudes [24], the impression that affected individuals are dangerous [25, 26] and the desire to avoid contact with them [16, 25, 27, 28].

Regarding psychosocial explanations, mixed results were also reported. While some studies suggest that psychosocial causal beliefs can reduce the desire for social distance [16, 24, 29] and stigmatising attitudes [30], others suggest that psychosocial causal beliefs are associated with the impression that affected people are more violent (dangerous) than people without depression [31].

Causal beliefs for depression related to lifestyle could lead to more stigmatisation, as lifestyle behaviour can be changed and thus a person might be considered responsible for the development of depression. According to the attribution theory, this then leads to more blame and fewer positive emotions [19, 20]. The causal beliefs that depression is a consequence of character weakness, lack of willpower and a wrong lifestyle are associated with lower social acceptance towards depressed individuals [22] and more desire for social distance [16, 27, 32]. Most studies examining lifestyle factors in the context of causal beliefs for depression use stigmatising statements such as “weakness of character” in their lifestyle measures. It is therefore not surprising that there are high correlations between scores on these items with stigma. In our opinion, there is a lack of studies phrasing lifestyle related items in a more neutral manner.

The above findings relate to personal stigma. In contrast to this type of stigma, which express people’s own stigma towards depression, perceived stigma describes peoples perception of others’ negative attitudes towards depression [7]. Perceived stigma can have a strong impact on help-seeking behaviour, as people expect to be exposed to negative evaluations from others. A study by Barney and Colleague’s [33] found that many subjects would feel embarrassed seeking professional help and believed that other people would react negatively to them if they sought such help. Self-embarrassment and the expectation of negative reactions by others reduced the likelihood of subjects to seek professional help.

To date, there are few studies examining perceived stigma with respect to causal beliefs. Nieuwsma & Pepper [34] found no significant relationship between perceived stigma and biogenetic or psychosocial causal beliefs. In other studies, endorsement of biogenetic causal beliefs was associated with greater perceived stigma of depression [35] and a higher number of perceived negative reactions towards people with schizophrenia, whereas psychosocial causal beliefs were unrelated to perceived discrimination against people with schizophrenia [36].

When studying perceived and personal stigma, personal contact with people with depression is likely to impact stigma [37–39]. People with depression and their relatives reported significant lower personal stigma than people without personal contact to someone affected [38, 39]. This finding could be related to an increased knowledge about the disease, which predicts lower personal stigma itself [37]. In contrast, individuals with more contact to people with depression showed higher levels of perceived stigma [38, 39]. It is possible that individuals with more exposure to depression have had more experiences with stigmatising attitudes and are therefore more aware of them. Since contact with depression has an impact on personal and perceived stigma, it is possible that contact as a moderator has an influence on the effect of causal beliefs on stigma.

The studies referred to, are mostly based on representative population surveys with between 1,400 and 6,000 participants. The explanatory power of the models and the observed effect sizes regarding causal beliefs were rather small, indicate that causal beliefs are only one factor among others that predict stigma or components of stigma. Nevertheless, it is of great importance to further investigate stigma and stigma-related factors. Understanding how factors such as beliefs about the aetiology of depression influence stigma will inform how to best communicate information on depression to the general public and to special target groups. Although effect sizes tend to be small, even small impacts can lead to worthwhile gains in a public health context that affects large numbers of people. Reducing stigma in the general population could improve the future situation of people with depression as they might experience fewer negative reactions and show more help-seeking behaviours.

### Objectives

Based on a representative survey of the adult population in Germany (“Deutschland-Barometer Depression 2018”) this study aimed to analyse:

1. relationships between (a) biogenetic, (b) psychosocial causal beliefs, (c) lifestyle causal beliefs and personal as well as perceived stigma.
2. whether personal contact with people with depression during ones lifespan moderates the associations between causal beliefs and perceived as well as personal stigma.

## Methods

### Survey

This publication uses data from the 2018 edition of the “Deutschland-Barometer Depression”, a representative survey of German adults on opinions and attitudes towards depression. The study was conducted within the cooperation between the German Depression Foundation and Deutsche Bahn Stiftung gGmbH.

### Sample

The online survey was conducted by Respondi (www.respondi.com), a market research company and panel provider, which is certified according to the internationally recognized ISO 26,362 standard. Sampling was stratified for age (18–69 years), gender (male/female) and place of residence of the respondents, resulting in a sample matching the general population for these characteristics. Concerning place of residence, we used Nielsen areas in order to best represent the German population. A Nielsen area comprises of one or more German federal states with as similar an economic situation as possible. A total of 7,259 panel members responded to the invitation to the survey. Of these, 141 (1.94%) were excluded because they did not fit the target group, 1,228 (16.91%) could not participate since the predefined quota was already accomplished and 357 (4.91%) did not complete the questionnaire. A total of 533 (7.34%) responders did not pass the quality standards applied by Respondi. The final sample comprised *N* = 5,000 participants.

### Measures

For the present purpose, only selected parts of the more comprehensive “Deutschland-Barometer Depression” survey were used. At the start of the survey, the participants were informed that the survey was about opinions and attitudes towards depression in the German population. They were first asked about their knowledge on the topic of depression, including an assessment of possible causes of depression. Then they were asked about their personal contact with the topic of depression. After, the participants completed a questionnaire on depression stigma, followed by further questions on treatment options for depression. The latter will not be used in the present analyses. For the exact wording of the items, see supplementary material.

#### Causal beliefs

To measure causal beliefs of depression, we presented participants a list of 13 possible beliefs. Each listed belief had to be rated on a four-point scale anchored by 1 = “very relevant” and 4 = “not relevant at all”. The list was developed based on a German population survey assessing knowledge about and attitudes towards depression by the competence network “Depression and Suicidality” (funded by the Federal Ministry of Education and Research) [40]. For our analyses, one item (“character weakness”) was removed because this is already a stigmatizing statement and there is an item with similar wording in the Depression Stigma Scale, which we used to survey personal and perceived stigma (see below).

#### Contact group

A multiple-choice item was used to group the sample according to the degree of proximity of respondents to people suffering from depression: “*Have you already come into contact with the illness depression?*” Participants could then choose one or more of the following options: (A) *Yes, I have already been diagnosed with depression once.* (B) *Yes, I think I have already had depression myself, but no diagnosis has been made.* (C) *Yes, a relative or friend has already been diagnosed with depression.* (D) *Yes, I treat/counsel people with depression.* If none of these options were considered applicable, participants could choose option (E) *No, I have no direct connection to the topic of depression.* These choices were conceptualised as a “contact group” for the present analysis. The variable comprises five levels: (1) persons who treat or advise affected individuals, (2) affected persons with a diagnosis of depression, (3) affected persons without a diagnosis of depression, (4) people close to a person with depression, and (5) people without a direct relationship to a person with depression. Level 1 indicates the closest contact with depression, while level 5 corresponds to no contact. If individuals chose more than one contact group option, the option describing a closer contact was chosen. For the current analysis, practitioners were defined as the group with closest contact to depression as they are experts for depression who work and interact with multiple people with depression and also have the most well-grounded knowledge about depression.

#### Stigma

The Depression Stigma Scale (DSS) [7] is the validated gold standard for assessing stigma. It consists of two subscales, each containing nine items for personal stigma and for perceived stigma. Both subscales range from 0 to 36. The subscale for perceived stigma measures how respondents rate other peoples’ attitudes towards depression. The subscale for personal stigma, on the other hand, reflects the respondents’ personal attitudes towards depression. The response scales range from 4 = “strongly agree” to 0 = “strongly disagree”. A high sum score corresponds to high stigma. The DSS has shown acceptable to good test-retest reliability and internal consistency (Cronbach’s Alpha for total stigma, personal stigma and perceived stigma respectively 0.78, 0.76 and 0.82) [39]. In the current sample, Cronbach’s Alpha for total stigma, personal stigma and perceived stigma was 0.85, 0.82 and 0.90. As part of the OSPI-Europe study [41], the DSS was translated into German and was made available for our study. The translation was conducted via translation and back translation procedure.

### Statistical analyses

Answers to the items about causal beliefs for depression were entered into an explorative principal-component factor analysis. The factor scores then formed the basis for further calculations. The explorative principal-component factor analysis with oblique rotation (direct oblimin) was conducted for 12 items of the list. The oblique rotation was chosen because it was assumed that different factors might be correlated with each other. The Kaiser–Meyer–Olkin measure verified the sampling adequacy for the analysis, KMO = 0.83 [42]. Bartlett’s test of sphericity χ² (66) = 19745.07, p < .001, indicated that correlations between items were sufficiently large for principal-component factor analysis. An initial analysis was run to obtain eigenvalues for each factor in the data. Three factors had eigenvalues over Kaiser’s criterion of 1 and in combination explained 60.00% of the variance. The table for the factor loadings after rotation can be found in Table S1 in the Supplementary Material. The items that cluster on the same factors suggest that factor 1 represented psychosocial causal beliefs, factor 2 represented lifestyle causal beliefs, and factor 3 biogenetic causal beliefs. For our analyses, we reversed the factor scores, with higher scores indicating higher agreement with the respective explanatory approach.

Multiple Regressions were performed with contact level and the factor scores of causal beliefs as predictor variables for the personal stigma subscale (model 1) as well as perceived stigma subscale (model 2) (sum scores) as dependent variables. The reference category for contact levels was set to be unaffected person, because this was the contact level with the least contact with persons with depression. The other contact levels were each compared with this reference category. Moderation analyses were run to determine whether the interaction between causal beliefs and contact level significantly predicted stigma. We calculated Cohen´s *f²* as the effect size [43]. In order to obtain this, we set the explained variance including the respective predictor in relation to the explained variance excluding the respective predictor. Age and gender were added as covariates to the models.

Statistical analyses were performed using SPSS 27 [44] and R-statistics [45]. The significance level was set at α = 0.05.

## Results

Table 1 shows the demographic characteristics of the total sample as well as separately for the five subsamples according to personal contact with depression.

**Table 1:** Respondents’ characteristics according to contact with depression

		Total	Practitioner	Affected (diagnosed)	Affected (undiagnosed)	Relatives	Unaffected
	*n* (%)	5000 (100)	144 (2.9)	1049 (21.0)	796 (16.0)	1315 (26.0)	1696 (33.9)
Gender							
male	*n* (%)	2520 (50.4)	62 (43.0)	445 (42.4)	398 (50.0)	704 (46.4)	1004 (59.2)
female	*n* (%)	2480 (49.6)	82 (57.0)	604 (57.6)	398 (50.0)	704 (53.5)	692 (40.8)
Age							
18–29 y	*n* (%)	1040 (20.8)	52 (36.1)	122 (11.6)	220 (27.6)	309 (23.5)	337 (19.9)
30–39 y	*n* (%)	910 (18.2)	28 (19.4)	165 (15.7)	146 (19.7)	267 (20.3)	304 (17.9)
40–49 y	*n* (%)	990 (19.8)	26 (18.1)	249 (23.7)	157 (21.0)	233 (17.7)	325 (19.2)
50–59 y	*n* (%)	1180 (23.6)	26 (18.1)	322 (30.7)	167 (21.0)	277 (21.1)	388 (22.9)
60–69 y	*n* (%)	880 (17.6)	12 (08.3)	191 (18.2)	106 (13.3)	229 (17.4)	342 (20.2)

### Associations with personal and perceived stigma

The total sample had a mean score of 11.13 on the personal stigma subscale (range 0–36). The perceived stigma values are much higher (mean score: 20.21; range 0–36).

The results of the multiple regression analyses are shown in Tables 2 and 3. The predictors accounted for 20% of the variance in the personal stigma score and 4% of variance in the perceived stigma score.

**Table 2:** Model 1: Summary of multiple regression results of personal stigma (*N* = 5,000, *R*^2^ = 0.20)

	B	*p*	*95% CI*
Constant	12.10	< 0.001***	[11.62, 12.56]
Factor 1 (Psychosocial)	-0.88	< 0.001***	[-1.12, -0.64]
Factor 2 (Lifestyle)	1.36	< 0.001***	[1.09, 1.64]
Factor 3 (Biogenetic)	-0.38	0.006**	[-0.65, -0.11]
Group: Affected (Diagnosed)	-4.95	< 0.001***	[-5.39, -4.51]
Group: Affected (Undiagnosed)	-1.21	< 0.001***	[-1.68, -0.74]
Group: Relatives	-2.29	< 0.001***	[-2.70, -1.89]
Group: Practitioner	-3.36	< 0.001***	[-4.33, -2.39]
Age Group	0.16	0.005**	[0.05, 0.27]
Gender (male)	1.00	< 0.001***	[0.69, 1.31]

*Notes*. *R*^*2*^ = *explained variance of the model*; B = *unstandardized coefficients*; *p* = p-Value

*** result is significant (*p* < .001); ** result is significant (p < .010)

**Table 3:** Model 2: Summary of multiple regression results of perceived stigma (N = 5,000, *R*^2^ = 0.04)

	B	*p*	*95%CI*
Constant	21.75	< 0.001***	[20.73, 22.42]
Factor 1 (Psychosocial)	0.69	< 0.001***	[0.38, 0.99]
Factor 2 (Lifestyle)	0.46	0.011*	[0.10, 0.81]
Factor 3 (Biogenetic)	-0.04	0.982	[-0.35, 0.34]
Group: Affected (Diagnosed)	0.85	0.003**	[0.29, 1.42]
Group: Affected (Undiagnosed)	0.41	0.179	[-0.19, 1.02]
Group: Relatives	-0.13	0.612	[-0.65, 0.38]
Group: Practitioner	-0.75	0.235	[-1.99, 0.49]
Age Group	-0.61	< 0.001***	[-0.74, -0.46]
Gender (male)	0.17	0.394	[-0.22, 0.57]

*Notes*. *R*^*2*^ = *explained variance of the model*; B = *unstandardized coefficients*; *p* = p-Value;

*** result is significant (*p* < .001); ** result is significant (p < .010); * result is significant (*p* < .050)

#### Associations between causal beliefs for depression regarding personal and perceived stigma

All causal beliefs were statistically significant predictors of personal stigma (all *ps < .*010) (see Table 2). Lifestyle causal beliefs (*f²* = 0.07) was positively related to personal stigma, whereas the psychosocial (*f²* = 0.02) and biogenetic causal beliefs (*f²* = 0.01) were negatively related to personal stigma. Thus, individuals who shared lifestyle causal beliefs tended to have higher levels of personal stigma, while individuals with stronger psychosocial and biogenetic causal beliefs tended to have lower levels of personal stigma.

As displayed in Table 3, only the factors psychosocial causal beliefs (p < .001) and lifestyle causal beliefs (p = .011) were significant predictors of perceived stigma. Thus, biogenetic causal beliefs were not associated with perceived stigma. Stronger lifestyle (*f²* = 0.01) and psychosocial causal beliefs (*f²* = 0.01) were related to higher perceived stigma.

#### Associations between contact with depression regarding personal and perceived stigma

Regarding personal stigma, each contact group differed significantly from the unaffected group (all *ps <* 0.001) (see Table 2). All other contact groups had significantly lower stigma scores than the unaffected group. The regression coefficients in Table 2 show that affected people with diagnosis had the lowest personal stigma. People without a direct exposure to depression had the highest personal stigma. The personal stigma of those affected without a diagnosis was also comparatively high.

Only the contact group affected (diagnosed) was statistically significant from the group without contact with depression (p = .003) regarding perceived stigma (see Table 3). The regression coefficients in Table 3 show that the perceived stigma scores were highest in the two contact groups affected.

#### Moderation effect between causal beliefs and personal as well as perceived stigma by contact levels

There was a statistically significant positive interaction effect between psychosocial causal beliefs and the contact group relatives (b = 0.418, t(4978) = 2.067, p = .039) regarding personal stigma. Furthermore on a descriptive level, there was a trend for a negative Interaction of biogenetic causal beliefs and contact group relatives (b = -0.399, *t*(4978) = -1.947, *p* = .051). The contact group of relatives thus benefit not so strongly from psychosocial causal beliefs but rather from biogenetic causal beliefs regarding personal stigma.

**Fig. 1:**
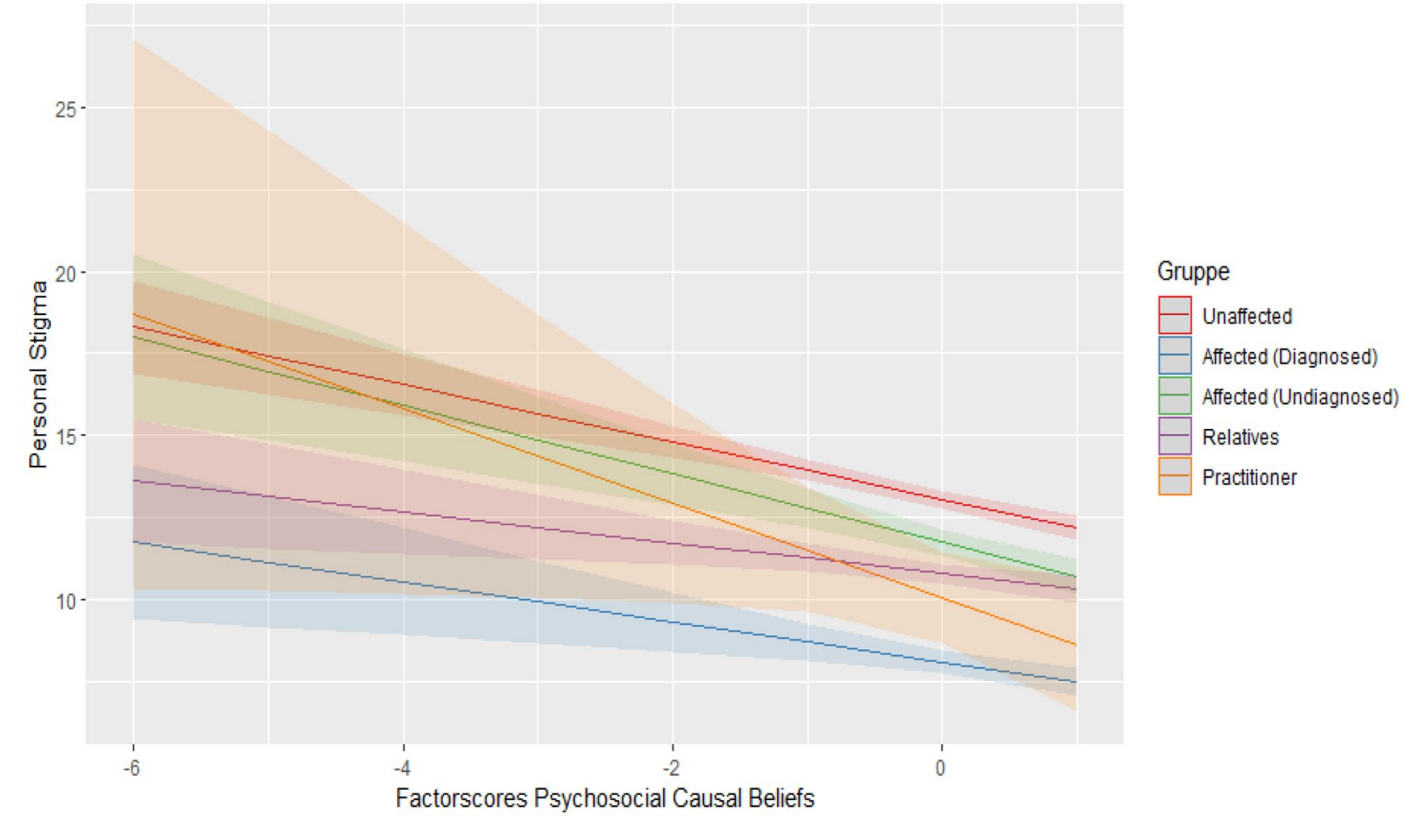
Interaction between psychosocial causal beliefs and contact groups regarding personal stigma

**Fig. 2:**
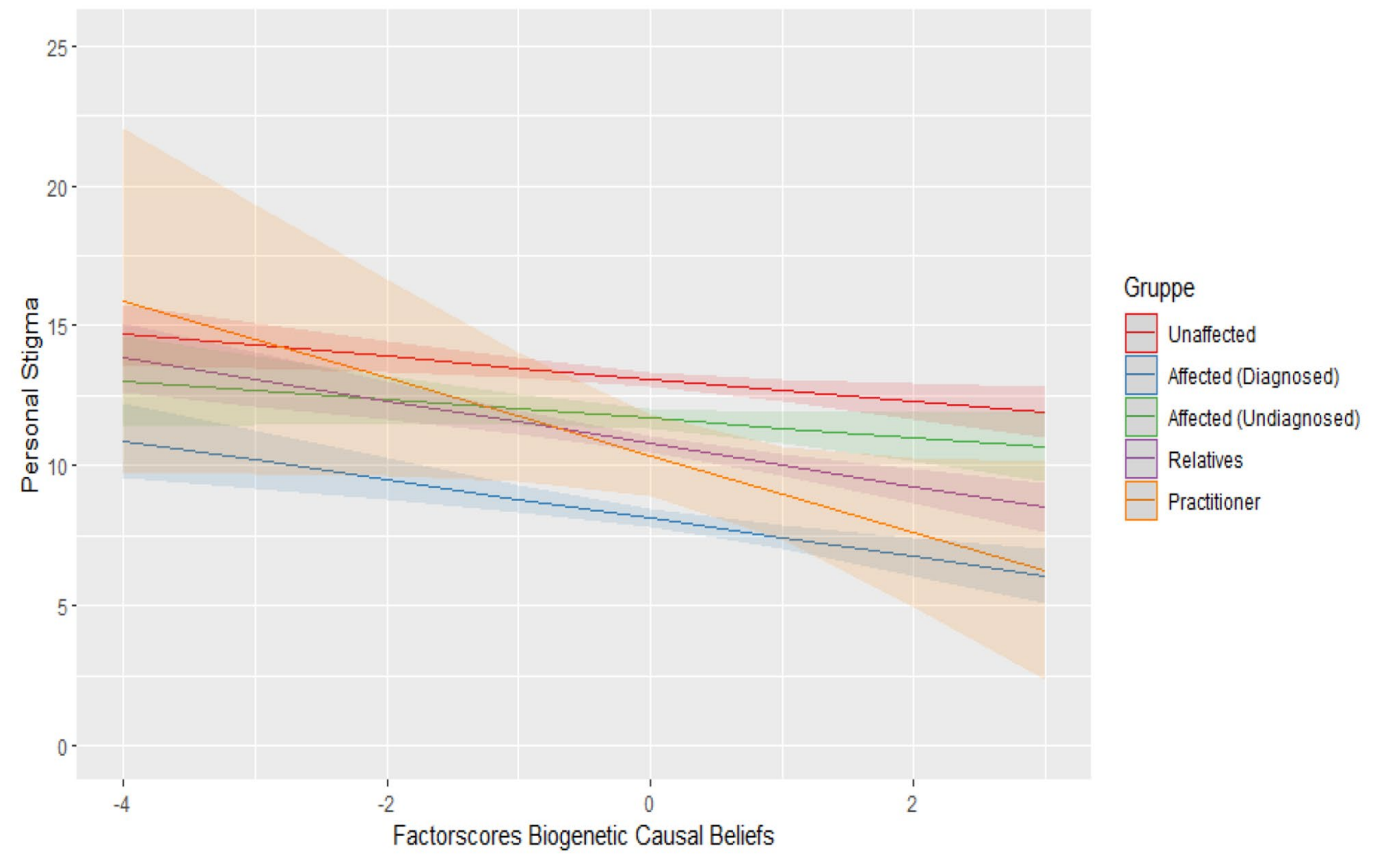
Interaction between biogenetic causal beliefs and contact groups regarding personal stigma

**Fig. 3:**
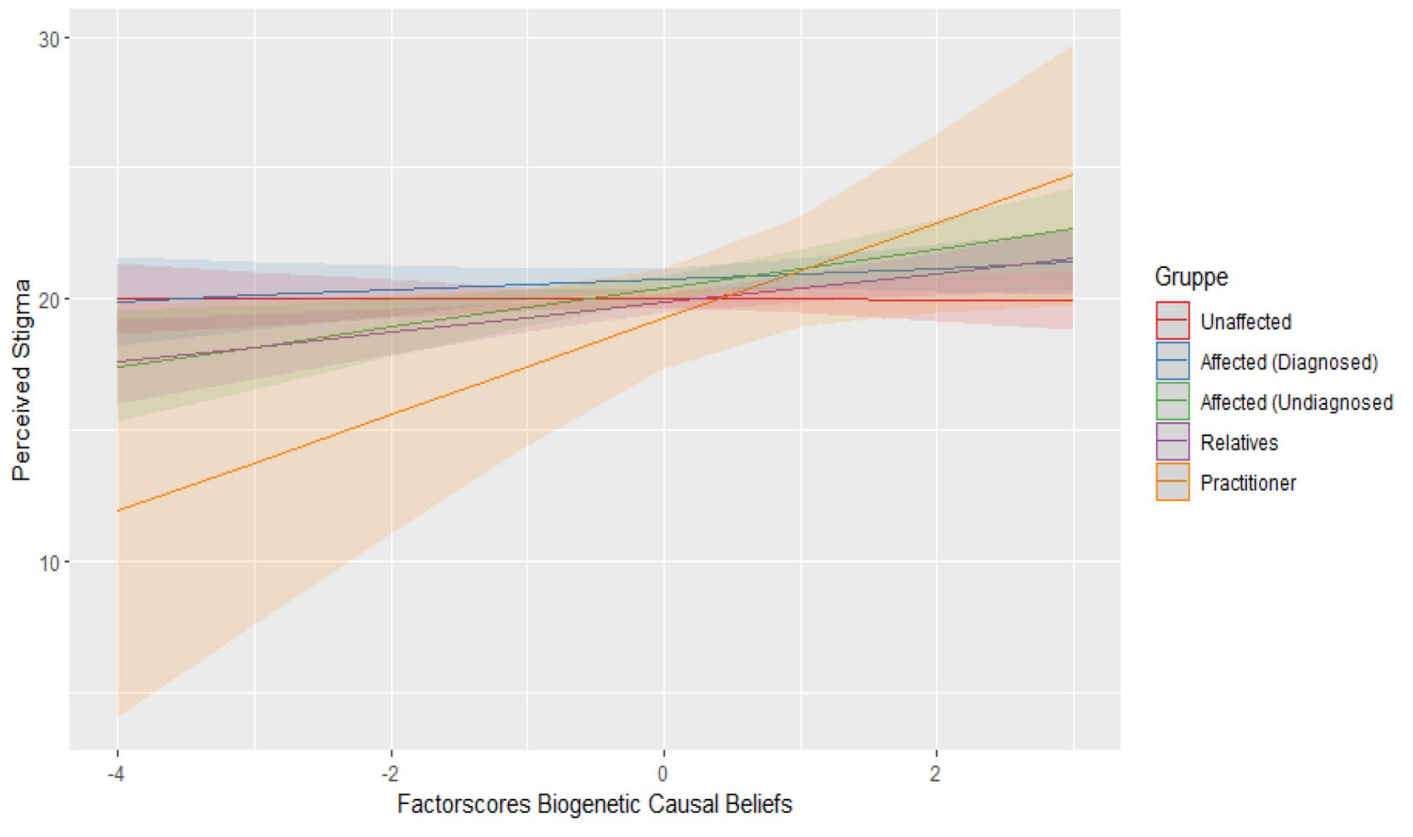
Interaction between biogenetic causal beliefs and contact groups regarding perceived stigma

Regarding perceived stigma, there was a statistically significant positive interaction of biogenetic causal beliefs and contact group practitioner (b = 2.252, *t*(4978) = 3.550, *p* < .001) and of biogenetic causal beliefs and contact group affected without diagnosis (b = 0.773, *t*(4978) = 2.421, *p* = .016). Therefore, the biogenetic approach is associated with higher stigma within contact groups of undiagnosed affected and practitioners. Figures 1, 2 and 3 show the mentioned interactions effects while holding constant the other predictor variables.

## Discussion

The present analysis examined causal beliefs for depression in relation to personal and perceived stigma. It was also examined whether the relationship between causal beliefs and stigma was moderated by the contact group. For the analyses, data from a representative sample of 5,000 people from across Germany were evaluated.

### Associations of causal beliefs for depression with personal and perceived stigma

People who were more likely to have biogenetic causal beliefs scored 0.38 points lower on the personal stigma scale (range 0–36) and persons who rather agreed with the psychosocial causal beliefs even scored 0.89 lower. This result is inconsistent with Colman and Collegues [24] who found that biogenetic causal beliefs were associated with more stigmatising attitudes and psychosocial beliefs with fewer. However consistent with Schnittker [21], who found positive associations of psychosocial and biogenetic causal beliefs and social acceptance of people with depression. The results imply that models to explain the causes of depression based on the interaction between biogenetic and psychosocial components, as is the case with the diathesis-stress model [46], are also effective in reducing stigma.

People who tended to hold lifestyle causal beliefs had a 1.36 higher personal stigma score. This finding is consistent with Cleveland and Collegues [16], who found that “personal causes” such as wrong lifestyle, poor nutrition and weakness of character, are associated with a stronger desire for social distance. Although we used less judgmental wording and thus fewer stigmatizing items, we also found this effect in our study. According to attribution theory [20], people who strongly agree with these causal beliefs, which relates to behavioural patterns, may believe that people with depression are largely in control of their illness and therefore can be blamed for it [47]. Accountability or blame for depression tends to contribute to higher personal stigma.

Regarding perceived stigma, both lifestyle causal beliefs and the psychosocial causal beliefs were associated with higher stigma. In previous studies, there was no significant relationship between psychosocial causal beliefs and perceived stigma [34, 36]. However, Nieuwsma and Pepper found a non-significant positive trend and, in line with our results, no association between biogenetic causal beliefs and perceived stigma [34].

### Associations between contact with depression and personal as well as perceived stigma

Our finding that the group with the least contact with depression has the highest personal stigma scores has also been found in other studies [37–39]. This could be due to the fact that this group has had the least exposure to the topic, has no opportunity to have corrective experiences and has had the least information about depression. Furthermore, it is worth highlighting that affected people without diagnosis have the second highest stigma scores. Receiving a diagnosis requires seeking help from a physician or therapist. It is possible that those affected have not received a diagnosis because their negative attitudes towards depression have prevented them from seeking help [48]. Further, they could not profit from the expert knowledge of a practitioner that might have the potential to reduce stigma.

In regard to perceived stigma, the group of diagnosed affected had significantly higher scores than those who had no contact with depression. This finding is in line with previous research [38, 39]. One reason could be that those affected may be more sensitised to the topic and might be confronted with stigmatizing comments in their everyday life.

### Moderation effect between causal beliefs and personal as well as perceived stigma by contact levels

The effects of causal beliefs on personal and perceived stigma are generally not moderated by the level of contact. However, there are moderation effects of the contact group regarding biogenetic causal beliefs.

Friends or family members of affected people did not benefit as strongly from psychosocial explanatory models. There is a non-significant trend that these individuals are rather benefiting from biological explanations of personal stigma. These results suggest that it is be important to educate especially about biogenetic aspects of the development of depression in anti-stigma campaigns targeting relatives of people with depression.

The biogenetic approach is associated with higher perceived stigma within the contact groups undiagnosed affected and practitioners. People who think they have suffered from depression during their lifespan but have not been diagnosed, and who agree with a biogenetic causal model, are therefore more likely to believe that stigmatising attitudes are widespread in the population. However, these are people who assume they have had depression. It is not known how many of them actually had depression. These findings suggest that the perceived stigma of people who think they have depression but are not in treatment could be reduced by educating them about other explanatory models, too (e.g. psychosocial), in anti-stigma campaigns for this target group. This could increase help-seeking behaviour by reducing worries about stigmatization in society. However, the results regarding perceived stigma must be interpreted very cautiously, as the variance explained is rather small.

### Interpretation of results

Predictors of personal stigma explained 20% of the variance. This represents a medium to high explanation of variance [49]. Predictors of perceived stigma explained 4% of the variance. This value is considered as low explained variance and an indicator of other existing variables that have a higher explanatory power. Furthermore, the effect sizes of the causal belief predictors are small. Lifestyle causal beliefs have the largest effect with *f²* = 0.07, which corresponds to a small effect according to Cohen (0.02 = small; 0.15 = medium; 0.35 = strong) [49]. Considering the unstandardised beta coefficients in the model for personal stigma, these are also relatively small for the causal beliefs (range 0.38–1.36) compared to the contact groups (range 1.21–4.95). However, Dardas and colleagues who also used the depression stigma scale also found betas between 0.4 and 0.9 for the causal beliefs [50]. Griffith and colleagues [39] found betas between 0.15 and 2.37 for various predictors. Thus, the effect sizes we found are within the usual range of this research field.

### Strengths and limitations

The lack of agreement on the concept of stigma presents a methodological challenge to build on existing stigma research [51]. By using the Depression Stigma Scale (DSS) as an internationally validated main instrument [52] some of these problems could be avoided. The DSS aims to specifically measure as many stigma components as possible that could play a role in depression and thus to comprehensively assess the stigma concept. The term “stigma” was not mentioned in the Deutschland-Barometer to avoid that participants’ subjective opinions of stigma could impact their responses. Another strength of the study is the large number of respondents (N = 5,000) leading to high power which allows to detect even small effects. In addition, the sample is representative of the German population, as the survey was based on the Nielsen Areas. Thus, the results can be generalised to the German population aged 18–69 years. The gender ratio of respondents was balanced (female: 48.4%). Furthermore, the risk of socially desirable answers was minimized: All answers were given anonymously and exclusively online. In face-to-face interviews [53] or telephone interviews [41] bias could be higher due to personal contact.

One limitation of the present study is that the causal beliefs survey instrument was not a validated questionnaire. Rather, it was a sample of listed causes from which subjects could choose. The factors representing different causal beliefs used in the main analyses were then extracted using an exploratory factor analysis. Furthermore, causal relationships cannot be established in this study. The ex post facto design allows only correlative conclusions, as all data were collected cross-sectionally at the same time point. Furthermore, only the age group of 18–69 years was surveyed. No conclusions can be drawn for other age groups. Moreover, self-stigma was not measured in this study. It would be interesting to investigate the relationship between causal beliefs and self-stigma in future studies. In this study, only biogenetic, psychosocial, and lifestyle causal beliefs were included. There are also other types of beliefs, such as continuum beliefs [54] and fatalistic beliefs [55], which could be considered in future studies.

## Conclusions

Particular, biogenetic and psychosocial causal beliefs are both related to lower personal stigma scores. Lifestyle causal beliefs are associated with higher personal stigma. The current data suggests that anti-stigma campaigns should communicate that the cause of depression is not an unfavourable lifestyle. In general, education should be provided on biogenetic or psychosocial approaches. When addressing relatives of depressed patients, biogenetic explanations in particular should be explained. The personal stigma of relatives is of great interest, as relatives can support patients in coping with depression. However, it is important to note that causal beliefs are only one of many factors that impact on stigma.

## Electronic supplementary material

Below is the link to the electronic supplementary material.

Supplementary Material 1

## Data Availability

The datasets used and analysed during the current study available from the corresponding author on reasonable request.

## References

[1] Ferrari AJ, Charlson FJ, Norman RE, Patten SB, Freedman G, Murray CJL (2013). Burden of depressive disorders by country, sex, age, and year: findings from the global burden of disease study 2010. PLoS Med.

[2] Bromet E, Andrade LH, Hwang I, Sampson NA, Alonso J, De Girolamo G (2011). Cross-national epidemiology of DSM-IV major depressive episode. BMC Med.

[3] Lim GY, Tam WW, Lu Y, Ho CS, Zhang MW, Ho RC (2018). Prevalence of depression in the community from 30 countries between 1994 and 2014. Sci Rep.

[4] Brohan E, Gauci D, Sartorius N, Thornicroft G, Group GS (2011). Self-stigma, empowerment and perceived discrimination among people with bipolar disorder or depression in 13 european countries: the GAMIAN–Europe study. J Affect Disord.

[5] Lasalvia A, Zoppei S, Van Bortel T, Bonetto C, Cristofalo D, Wahlbeck K, ASPEN/INDIGO Study Group (2013). Global pattern of experienced and anticipated discrimination reported by people with major depressive disorder: a cross-sectional survey. Lancet.

[6] Haslam N, Kvaale EP (2015). Biogenetic explanations of mental disorder: the mixed-blessings model. Curr Dir Psychol Sci.

[7] Griffiths KM, Christensen H, Jorm AF, Evans K, Groves C (2004). Effect of web-based depression literacy and cognitive–behavioural therapy interventions on stigmatising attitudes to depression: Randomised controlled trial. Br J Psychiatry.

[8] Arnaez JM. A Cross-sectional Analysis of Depression Stigma and Barriers to Seeking Mental Health Care: Evidence for Effect Modification by Depression Severity, Cultural Background, and Gender. Doctoral dissertation. Indiana University; 2018.

[9] Clement S, Schauman O, Graham T, Maggioni F, Evans-Lacko S, Bezborodovs N (2015). What is the impact of mental health-related stigma on help-seeking? A systematic review of quantitative and qualitative studies. Psychol Med.

[10] Evans-Lacko S, Brohan E, Mojtabai R, Thornicroft G (2012). Association between public views of mental illness and self-stigma among individuals with mental illness in 14 european countries. Psychol Med.

[11] Schomerus G, Stolzenburg S, Freitag S, Speerforck S, Janowitz D, Evans-Lacko S (2019). Stigma as a barrier to recognizing personal mental illness and seeking help: a prospective study among untreated persons with mental illness. Eur Arch Psychiatry Clin Neurosci.

[12] Hirschfeld RMA, Keller MB, Panico S, Arons BS, Barlow D, Davidoff F (1997). The National Depressive and Manic-Depressive Association consensus statement on the undertreatment of depression. JAMA.

[13] Corrigan PW, Watson AC (2002). The paradox of self-stigma and mental illness. Clin Psychol Sci Pract.

[14] Livingston JD, Boyd JE (2010). Correlates and consequences of internalized stigma for people living with mental illness: a systematic review and meta-analysis. Soc Sci Med.

[15] Breheny M (2007). Genetic attribution for schizophrenia, depression, and skin cancer: impact on social distance. NZ J Psychol.

[16] Cleveland H, Baumann A, Zäske H, Jänner M, Icks A, Gaebel W (2013). Association of lay beliefs about causes of depression with social distance. Acta Psychiatr Scand.

[17] Lee AA, Farrell NR, McKibbin CL, Deacon BJ (2016). Comparing treatment relevant etiological explanations for depression and social anxiety: Effects on self-stigmatizing attitudes. J Soc Clin Psychol.

[18] Mayville E, Penn DL (1998). Changing societal attitudes toward persons with severe mental illness. Cogn Behav Pract.

[19] Rush LL (1998). Affective reactions to multiple social stigmas. J Soc Psychol.

[20] Weiner, Perry RP, Magnusson J (1988). An attributional analysis of reactions to stigmas. J Pers Soc Psychol.

[21] Schnittker J (2008). An uncertain revolution: why the rise of a genetic model of mental illness has not increased tolerance. Soc Sci Med.

[22] Cook T, Wang J (2011). Causation beliefs and stigma against depression: results from a population-based study. J Affect Disord.

[23] Read J, Harré N (2001). The role of biological and genetic causal beliefs in the stigmatisation of’mental patients’. J Ment Heal.

[24] Colman L, Delaruelle K, Luypaert C, Verniest R, Bracke P (2021). Burdens in mental health recovery: causal beliefs and their relation to stigma and help seeking recommendations. Int J Soc Psychiatry.

[25] Schomeus G, Matschinger H, Angermeyer M (2014). Causal beliefs of the public and social acceptance of persons with mental illness: a comparative analysis of schizophrenia, depression and alcohol dependence. Psychol Med.

[26] Dietrich S, Matschinger H, Angermeyer MC (2006). The relationship between biogenetic causal explanations and social distance toward people with mental disorders: results from a population survey in Germany. Int J Soc Psychiatry.

[27] Dietrich S, Beck M, Bujantugs B, Kenzine D, Matschinger H, Angermeyer MC (2004). The relationship between public causal beliefs and social distance toward mentally ill people. Aust New Zeal J Psychiatry.

[28] von dem Knesebeck O, Angermeyer MC, Kofahl C, Makowski AC, Mnich E (2014). Education and the public’s desire for social distance from people with depression and schizophrenia: the contribution of emotional reactions and causal attributions. Int J Soc Psychiatry.

[29] Kermode M, Bowen K, Arole S, Pathare S, Jorm AF (2009). Attitudes to people with mental disorders: a mental health literacy survey in a rural area of Maharashtra, India. Soc Psychiatry Psychiatr Epidemiol.

[30] Read J, Law A (1999). The relationship of causal beliefs and contact with users of mental health services to attitudes to the’mentally ill’. Int J Soc Psychiatry.

[31] Goldstein B, Rosselli F (2003). Etiological paradigms of depression: the relationship between perceived causes, empowerment, treatment preferences, and stigma. J Ment Heal.

[32] Martin JK, Pescosolido BA, Tuch SA. Of fear and loathing: the role of’disturbing behavior,’labels, and causal attributions in shaping public attitudes toward people with mental illness. J Health Soc Behav. 2000;:208–23.

[33] Barney LJ, Griffiths KM, Jorm AF, Christensen H (2006). Stigma about depression and its impact on help-seeking intentions. Aust New Zeal J Psychiatry.

[34] Nieuwsma JA, Pepper CM (2010). How etiological explanations for depression impact perceptions of stigma, treatment effectiveness, and controllability of depression. J Ment Heal.

[35] Wadian S. The impact of stigma and etiological beliefs on willingness to seek treatment for depression: A comparison of undergraduate and behavioral sciences graduate Students. Doctoral Dissertation. Texas Tech University; 2013.

[36] Peluso ÉTP, Blay SL (2011). Public stigma and schizophrenia in São Paulo city. Brazilian J Psychiatry.

[37] Busby Grant J, Bruce CP, Batterham PJ (2016). Predictors of personal, perceived and self-stigma towards anxiety and depression. Epidemiol Psychiatr Sci.

[38] Calear AL, Griffiths KM, Christensen H (2011). Personal and perceived depression stigma in australian adolescents: magnitude and predictors. J Affect Disord.

[39] Griffiths KM, Christensen H, Jorm AF (2008). Predictors of depression stigma. BMC Psychiatry.

[40] Althaus D, Stefanek J, Hasford J, Hegerl U (2002). Wissensstand und Einstellungen der Allgemeinbevölkerung zu Symptomen, Ursachen und Behandlungsmöglichkeiten depressiver Erkrankungen. Nervenarzt.

[41] Coppens E, Van Audenhove Ch, Scheerder G, Arensman E, Coffey C, Costa S, Koburger N, Gottlebe K, Gusmão R, O’Connor R, Postuvan V, Sarchiapone M, Sisask M, Székely A, van der Feltz-Cornelis C, Hegerl U (2013). Public attitudes towards depression and help-seeking in four european countries baseline survey prior to the OSPI-Europe intervention. J Affect Disord.

[42] Field A. Discovering statistics using IBM SPSS statistics. sage; 2013.

[43] Selya AS, Rose JS, Dierker LC, Hedeker D, Mermelstein RJ (2012). A practical guide to calculating Cohen’sf 2, a measure of local effect size, from PROC MIXED. Front Psychol.

[44] Statistics IS, IBM, Corp. Released 2013. IBM SPSS Statistics for Windows, Version 22.0. Armonk, NY: IBM Corp. Google Search. 2013.

[45] Team RC. R: A language and environment for statistical computing. R Foundation for Statistical Computing, Vienna, Austria. http//www R-project org/. 2013.

[46] Monroe SM, Simons AD (1991). Diathesis-stress theories in the context of life stress research: implications for the depressive disorders. Psychol Bull.

[47] Weiner B. Judgments of responsibility: a foundation for a theory of social conduct. guilford Press; 1995.

[48] Eisenberg D, Downs MF, Golberstein E, Zivin K (2009). Stigma and help seeking for mental health among college students. Med Care Res Rev.

[49] Cohen J (1988). Statistical power analysis for the behavioral sciences.

[50] Dardas LA, Silva SG, Scott J, Gondwe KW, Smoski MJ, Noonan D (2018). Do beliefs about depression etiologies influence the type and severity of depression stigma? The case of arab adolescents. Perspect Psychiatr Care.

[51] Link BG, Phelan JC (2001). Conceptualizing stigma. Annu Rev Sociol.

[52] Boerema AM, van Zoonen K, Cuijpers P, Holtmaat CJM, Mokkink LB, Griffiths KM (2016). Psychometric properties of the Dutch Depression Stigma Scale (DSS) and associations with personal and perceived stigma in a depressed and community sample. PLoS ONE.

[53] Lanfredi M, Zoppei S, Ferrari C, Bonetto C, Van Bortel T, Thornicroft G (2015). Self-stigma as a mediator between social capital and empowerment among people with major depressive disorder in Europe: the ASPEN study. Eur Psychiatry.

[54] Peter L, Schindler S, Sander C, Schmidt S, Muehlan H, McLaren T (2021). Continuum beliefs and mental illness stigma: a systematic review and meta-analysis of correlation and intervention studies. Psychol Med.

[55] Caplan S, Paris M, Whittemore R, Desai M, Dixon J, Alvidrez J (2011). Correlates of religious, supernatural and psychosocial causal beliefs about depression among latino immigrants in primary care. Ment Health Relig Cult.

