# Supplementary material for: Lifestyle causal beliefs are associated with higher personal and perceived stigma regarding depressive disorders: results from a representative population survey
Source: BMC Psychiatry. 2023 Jun 8;23:414. doi: 10.1186/s12888-023-04907-5 (PMC10249268; doi:10.1186/s12888-023-04907-5)
Supplement: Supplementary file 1 — Supplementary Material 1 [file 12888_2023_4907_MOESM1_ESM.docx]

**Supplementary Material**

**Results of factor analysis**

Table S1: *Summary of exploratory principal-component factor analysis results for causal beliefs (N = 5,000)*

|  | Rotated Factor Loadings | | |
| --- | --- | --- | --- |
| Items* | Psychosocial | Lifestyle | Biogenetic |
| Stroke of fate | **.68** | -.02 | .18 |
| Strain at work | **.76** | .16 | -.07 |
| Information overload and permanent accessibility | **.59** | .28 | -.07 |
| Loneliness | **.74** | .01 | .10 |
| Problems in relationship with the partner | **.82** | .01 | .07 |
| Stress and excessive demands | **.81** | .09 | .01 |
| Conflicts at work / with colleagues | **.83** | .07 | .01 |
| Unfavourable lifestyle | .12 | **.62** | .01 |
| Unfavourable nutrition | -.01 | **.84** | .22 |
| Environmental toxins | .09 | **.79** | .23 |
| Brain metabolic disorder | -.01 | .30 | **.76** |
| Genetics | .12 | .07 | **.83** |
|  |  |  |  |
| Eigenvalues | 4.01 | 2.03 | 1.14 |
| % of variance | 33.44 | 16.94 | 9.50 |

Annotations:

*Question: How relevant to the development of depression do you consider the following causes to be?

Factor loadings over .50 appear in bold.

**Questionaire-Items (English translation)**

The 2018 edition of the "Deutschland-Barometer Depression" survey comprised of various items on different topics. Here, we present those items that have been used for the present analyses.

**Contact with depression**

***Are you already come into contact with the disease depression? (Multiple selection possible)***

O Yes, I have already been diagnosed with depression once.

O Yes, I think I have already had depression myself, but no diagnosis has been made.

O Yes, a relative or acquaintance has already been diagnosed with depression.

O Yes, I treat/counsel people with depression.

O No, I have no direct connection to the topic of depression.

# Causal beliefs for depression

# *How relevant to the development of depression do you consider the following causes to be?*

|  | very relevant | relevant | less relevant | Not at all relevant |
| --- | --- | --- | --- | --- |
| Unfavorable lifestyle |  |  |  |  |
| Brain metabolic disorder |  |  |  |  |
| Stroke of fate (e.g. death of a relative) |  |  |  |  |
| Genetics |  |  |  |  |
| Unfavorable nutrition |  |  |  |  |
| Environmental toxins |  |  |  |  |
| Strain at work |  |  |  |  |
| Weakness of character |  |  |  |  |
| Information overload and permanent accessibility |  |  |  |  |
| Loneliness |  |  |  |  |
| Problems in relationship with the partner |  |  |  |  |
| Conflicts at work / with colleagues |  |  |  |  |
| Stress and excessive demands |  |  |  |  |
